# Prediction of rehabilitation induced motor recovery after stroke using a multi-dimensional and multi-modal approach

**DOI:** 10.3389/fnagi.2023.1205063

**Published:** 2023-07-04

**Authors:** Silvia Salvalaggio, Andrea Turolla, Martina Andò, Rita Barresi, Francesca Burgio, Pierpaolo Busan, Anna Maria Cortese, Daniela D’Imperio, Laura Danesin, Giulio Ferrazzi, Lorenza Maistrello, Eleonora Mascotto, Ilaria Parrotta, Rachele Pezzetta, Elena Rigon, Anna Vedovato, Sara Zago, Marco Zorzi, Giorgio Arcara, Dante Mantini, Nicola Filippini

**Affiliations:** ^1^IRCCS San Camillo Hospital, Venice, Italy; ^2^Padova Neuroscience Center, Università degli Studi di Padova, Padua, Italy; ^3^Department of Biomedical and Neuromotor Sciences (DIBINEM), Alma Mater Studiorum – Università di Bologna, Bologna, Italy; ^4^Unit of Occupational Medicine, IRCCS Azienda Ospedaliero-Universitaria di Bologna, Bologna, Italy; ^5^Department of Rehabilitation Medicine, AULSS 3 Serenissima, Venice, Italy; ^6^Philips Healthcare, Milan, Italy; ^7^Department of Physical Medicine and Rehabilitation, Venice Hospital, Venice, Italy; ^8^General Hospital San Camillo of Treviso, Treviso, Italy; ^9^Department of General Psychology, University of Padova, Padua, Italy; ^10^Movement Control and Neuroplasticity Research Group, KU Leuven, Leuven, Belgium

**Keywords:** stroke, rehabilitation-induced recovery, prediction, prognosis, neurophysiology, neuroimaging, biomarkers, upper limb

## Abstract

**Background:**

Stroke is a debilitating disease affecting millions of people worldwide. Despite the survival rate has significantly increased over the years, many stroke survivors are left with severe impairments impacting their quality of life. Rehabilitation programs have proved to be successful in improving the recovery process. However, a reliable model of sensorimotor recovery and a clear identification of predictive markers of rehabilitation-induced recovery are still needed. This article introduces the cross-modality protocols designed to investigate the rehabilitation treatment’s effect in a group of stroke survivors.

**Methods/design:**

A total of 75 stroke patients, admitted at the IRCCS San Camillo rehabilitation Hospital in Venice (Italy), will be included in this study. Here, we describe the rehabilitation programs, clinical, neuropsychological, and physiological/imaging [including electroencephalography (EEG), transcranial magnetic stimulation (TMS), and magnetic resonance imaging (MRI) techniques] protocols set up for this study. Blood collection for the characterization of predictive biological biomarkers will also be taken. Measures derived from data acquired will be used as candidate predictors of motor recovery.

**Discussion/summary:**

The integration of cutting-edge physiological and imaging techniques, with clinical and cognitive assessment, dose of rehabilitation and biological variables will provide a unique opportunity to define a predictive model of recovery in stroke patients. Taken together, the data acquired in this project will help to define a model of rehabilitation induced sensorimotor recovery, with the final aim of developing personalized treatments promoting the greatest chance of recovery of the compromised functions.

## 1. Introduction

Stroke is a cerebrovascular disease representing the third cause of death in high socio-demographic countries (Institute for Health Metrics and Evaluation [IHME], 2018). Improvements in prevention and treatment of the acute stage have significantly increased the survival rate. However, stroke remains a leading cause of severe long-term motor disability, affecting the quality of life of stroke survivors, limiting their return to a normal life, and representing a burden for their families. Worldwide there are over 33 million stroke survivors most of whom suffer from long-term disability (Feigin and Krishnamurthi, 2010).

Upper limb (UL) impairment represents the most impacting long-term disability caused by stroke. Among stroke survivors, motor impairment can be related to different aspects of movement, such as motor planning, learning, and control (Pollock et al., 2014). The aim of rehabilitation-mediated recovery is twofold: (i) minimize sequelae and (ii) improve the recovery of the affected limb(s).

Despite important advances in the medical and physical rehabilitation fields, neurological rehabilitation still lacks a reliable physiological model of UL sensorimotor recovery after stroke. For example, the magnitude of the motor recovery, occurring after rehabilitation treatment, that can be effectively attributed to the rehabilitation process itself is still largely unclear. Moreover, a precise identification and characterization of the key markers to be used as predictive factors of rehabilitation-induced recovery is needed (Hayward et al., 2017).

To date, both in clinical trials and current clinical practice, many treatment methods and assessment tools are available for quantification of the final outcome. Alongside the traditional therapeutic approaches (generally referred as “Conventional Therapy,” CT) based on neurodevelopmental principles, the so-called “innovative approaches” (e.g., robotics and virtual reality – VR) emerged recently providing an augmented environment with reinforced feedback to the patient. These technology-based methods have become popular, although current evidence on their efficacy is still under investigation (Feigin and Krishnamurthi, 2010; Bernhardt et al., 2016).

Evidence suggests that spontaneous recovery expresses its maximum effect from 3 to 6 months (Bernhardt et al., 2017), nevertheless recent results demonstrated that rehabilitation interventions can promote clinically significant improvements of motor outcomes even after this sensitive window, especially if high dose of therapy is provided (Daly et al., 2019; Ward et al., 2019). Indeed, motor improvements in UL recovery were found to be achieved after 90–300 h of rehabilitation, even in the chronic phase (Daly et al., 2019; Ward et al., 2019). Moreover, data available in the literature also emphasize the importance of the so-called “active ingredients” of rehabilitation (i.e., the specific elements which are assumed to be responsible for the treatment effect), but which are often not defined, classified, or measured (Ward et al., 2019).

During motor performance, there is an integration between motor and cognitive components (Barrett and Muzaffar, 2014; D’Imperio et al., 2021). For example, the control of sensorimotor aspects of motor actions requires attentional and cognitive demands, especially when performing complex movements. Consequently, cognitive abilities, such as attention, may play a key role, particularly in individuals recovering from stroke, as suggested by several studies (McDowd et al., 2003; Krakauer, 2006; Shafizadeh et al., 2017; VanGilder et al., 2020). This indicates that the identification of cognitive skills capable of guiding and predicting motor recovery could help in “*a priori*” patients stratification based on different recovery potentials.

Alongside motor recovery, prediction of the optimal level of functional improvement to be expected, is also a critical aspect in the stroke rehabilitation field. Indeed, prediction may be a useful guidance for setting rehabilitation goals and monitoring patient’s achievements over time (Piscitelli et al., 2018). Up to date, literature has mainly focused on prognostic factors (i.e., considering spontaneous recovery) rather than predictive ones (i.e., considering response to a rehabilitative intervention) (Clark, 2008). Coupar et al. in a systematic review examined potential factors for predicting UL recovery, such as: (i) preserved conduction and anatomical integrity of the cortico-spinal tract (CST) confirmed by motor evoked potentials (MEPs), and fractional anisotropy (FA), respectively; (ii) preserved sensation function; (iii) strong Shoulder Abduction and Finger Extension (SAFE) (Coupar et al., 2012; Stinear et al., 2017b). However, these studies did not take into account whether patients received rehabilitation or the dosage and modalities used, therefore, the impact of therapy and/or dosage on the accuracy of predictions has not yet been evaluated (Coupar et al., 2012; Stinear et al., 2017b). One study suggested that the best predictors of response to robotic treatment were preserved CST integrity, great ipsilesional motor cortex activation, and great interhemispheric connectivity, although the dosage used was still relatively low and not investigated as a factor potentially influencing the recovery prediction (Burke Quinlan et al., 2015). Likewise, improvement of motor functions after stroke can be associated with some changes in brain activity and connectivity as measured with electroencephalography (EEG): on inter-regional synchronization of neural oscillations (Nicolo et al., 2015; Romeo et al., 2021), on the evoked responses as measured in an oddball task (Naatanen et al., 2012), or in the brain oscillations evoked during gamma entrainment (Pellegrino et al., 2019). Some recent results also suggest that the aperiodic component of the power spectrum can have an important prognostic role in brain damaged patients (Maschke et al., 2023).

With regards to recovery prediction, data from the literature suggest that specific algorithms can be used to provide an accurate prediction of UL functional recovery. These algorithms are mostly based on clinical, imaging (magnetic resonance imaging – MRI) and neurophysiological (MEPs, collected with transcranial magnetic stimulation – TMS) measures. However, the validity of such models has been investigated only with assessments performed within a time window from 2 to 11 days (i.e., 2, 3, 5, 9, and 11 days) and UL prediction recovery at 3 months (Stinear, 2017). Moreover, factors such as: (i) variability in time of patient transfer to rehabilitation facilities, (ii) absence or delay in the acquisition of timely specific information (i.e., MEPs, FA, and National Institute of Health Stroke Scale – NIHSS), and (iii) different phases of recovery (i.e., subacute and chronic) when patients join the rehabilitation care, represent some of the most common confounders that can severely affect the predictive accuracy of the algorithms (Smania et al., 2009; Stinear, 2010, 2017; Stinear et al., 2012, 2017a). If only clinical outcome measures are available, predicting the effect of true recovery due to “treatment” over time is virtually impossible. Indeed, standalone clinical measures do not allow the thorough interpretation of the mechanisms underlying UL functional recovery (Stinear et al., 2007). For these reasons, imaging and physiological techniques, such as functional magnetic resonance imaging (fMRI) and TMS, have been previously used to accrue information on motor-related brain regions functionality following stroke recovery (Allman et al., 2016; Smith and Stinear, 2016). Similarly, diffusion tensor imaging (DTI) technique can be used to determine the anatomo-histological integrity of motor-related white matter (WM) pathways (Stinear et al., 2007). Recent preliminary studies have also suggested that measuring plasma levels of specific miRNAs (small RNA molecules that act as regulators of cell development, proliferation, cycling, and differentiation), and determining the specific genetic profile of stroke patients could provide useful biologically derived predictive markers of functional recovery (Chang et al., 2016; Edwardson et al., 2018; Dimyan et al., 2022).

Here, we report the protocol of the study “NeuroPro” (Investigation of NEUROphysiological substrates of UL sensorimotor impairment after stroke and PROgnosis of rehabilitation-induced recovery of motor function: longitudinal study), which is a longitudinal study employing a multi-dimensional and multi-modal approach to evaluate motor function and predict recovery in stroke inpatients in a clinical setting. We will combine clinical, biological, neurophysiological/imaging, neuropsychological, and rehabilitation measures with the final aim of developing a multidimensional predictive model of motor recovery after stroke. Whereas clinical, neuropsychological, and rehabilitation measures are usually acquired in ecological clinical settings, the possibility of performing this study in a rehabilitation research center, with both clinical and research compounds in the same institute, will allow a detailed characterization of participants (e.g., clinical profiling, operationalization of pharmacological treatment, and individual frailty levels). Moreover, the access to neurophysiological/imaging and biological facilities, will grant us the possibility to acquire a set of specific measures to develop a comprehensive and sensitive model of motor recovery, after rehabilitation treatments in stroke survivors.

## 2. Objectives

The overarching objective of this project is to identify a set of variables, from a wide range of assessment modalities, which can reliably predict UL motor recovery after stroke, in patients undergoing rehabilitation in a clinical setting. This will help us to: (i) examine clinical features (e.g., behavioral, physiological, neural, and biological) associated with rehabilitation-induced recovery for identifying best responders to UL behavioral intervention, for rehabilitation after stroke, (ii) understand which modalities are the most suitable for a specific patient and the respective optimal dosage, and (iii) establish most effective times from onset of the acute event for modalities referral.

The richness of data acquired will also contribute to add new evidence on the predictive value of some emerging motor features (e.g., SAFE), biological markers (e.g., miR-941 levels), or physiological measures (e.g., EEG aperiodic parameters), described in greater detail in section “3. Materials and methods,” with the potential of being translated into current clinical practice.

## 3. Materials and methods

For a full and comprehensive reporting of the present study, the *transparent reporting of a multivariable prediction model for individual prognosis or diagnosis* (TRIPOD) will be used (Collins et al., 2015).

### 3.1. Funding, ethics, and data access

The study *NeuroPro* is partially funded by the RF-2018-12366899 “Brain connectivity measured with high-density electroencephalography: a novel neurodiagnostic tool for stroke” and GR-2018-12366092 “Assessment and treatment of communicative pragmatic abilities in neurological and psychiatric disorders: feasibility and clinical efficacy” grants by the Italian Ministry of Health.

Ethical approval was granted by the “Comitato etico per la Sperimentazione Clinica (CESC) della Provincia di Venezia e IRCCS San Camillo” (Prot. 1375/IRCCS San Camillo). The protocol has been registered on ClinicalTrials.gov (NCT05423119). Data collection for this study started in August 2021 and will be completed by February 2024. The study is carried out according to the Declaration of Helsinki. Written informed consent is obtained from all patients. Data are anonymized before being processed and stored on password protected computers or in protected areas within the IRCCS San Camillo Hospital infrastructures. All personal information (i.e., names and addresses) is stored separately in locked-filing cabinets. Only authorized personnel have access to patients’ data.

Data can be accessed upon request to the IRCCS San Camillo Hospital according to GDPR and Italian regulations for the privacy of biomedical data. A submission to the local ethical committee and informed consent from the participants may be required.

### 3.2. Participants’ recruitment and study design

#### 3.2.1. Participants

Study participants are recruited among stroke survivors admitted to a period of intensive neurorehabilitation treatment at the IRCCS San Camillo Hospital in Venice, Italy.

Inclusion criteria are: (1) age ≥18 years old; and (2) first ever cortical-subcortical, supratentorial ischemic or hemorrhagic, unilateral stroke, based on medical records.

Exclusion criteria are: (1) bilateral or pure cerebellar lesion; (2) presence of non-stabilized fractures; (3) diagnosis of other neurological and/or psychiatric disorder; (4) unstable medical condition (e.g., heart failure, untreated seizures, and psychiatric comorbidities); (5) any other relevant UL musculoskeletal impairment both before and after stroke, hampering assessment; and (6) inability to provide informed consent. Specific inclusion criteria for imaging analyses are (1) distinguishable lesion in FLAIR and (2) unilateral hemisphere lesion, while exclusion criteria are (1) bilateral lesion and (2) non-distinguishable lesion in FLAIR.

Specific exclusion criteria related to the instrumental technology (i.e., EEG, MRI, and TMS) employed in this project will be detailed in each specific section.

#### 3.2.2. Sample size calculation

The sample size was calculated with regard to the main motor outcome assessing UL function (Fugl-Meyer Assessment for Upper Extremity – FMA-UE). From published data on the same cohort study design of stroke survivors admitted at the IRCCS San Camillo Hospital (Salvalaggio et al., 2023), and undergoing the same rehabilitative treatments described in this protocol, is expected that UL function improves with moderate standardized effect (Cohen’s *d* = 0.45), according to FMA-UE. Assuming an equivalent effect size *f* = 0.225, for repeated measures, within factors multivariate analysis of variance (MANOVA) design (Chen and Chen, 2010), in one group with two measurements correlating 0.5, given α = 0.05 and 1-β = 0.90, a total recruitment of 54 consecutive subjects would be needed. Considering a drop-out rate of 40%, a final number of 75 patients will be considered sufficient to conclude the study.

#### 3.2.3. Study design

The study is a longitudinal cohort study. Every participant undergoes rehabilitation treatment according to the individualized plan agreed between the neurorehabilitation team and the same patient. Participation in the study does not result in the exclusion or reduction of ordinary treatment for the study-subjects. Full assessment is carried out before and after rehabilitation, according to the following scheme:

1. Initial assessment (T0): the participant undergoes clinical (motor and cognitive) assessments, blood sampling and instrumental investigations (i.e., imaging, neurophysiology, and electrophysiology), within 10 days from admission.
2. Treatment during hospitalization: every participant undergoes a motor rehabilitation program consisting at minimum of 1 h/day of conventional therapy (CT) and one or more hours of other modalities such as technology devices (i.e., robotics and VR) for upper and lower limb or occupational therapy (OT) UL specific, according to the individual rehabilitation project agreed with the rehabilitation team (e.g., physiotherapist and medical doctor) and tailored on patients’ needs. Each session is adapted to the patient’s clinical condition and motor ability to perform exercises, accomplishing any harm that may occur (e.g., patients referring shoulder pain and high spasticity). Overall, rehabilitation treatment lasts at least 4 consecutive weeks, with an average duration of 8 weeks.
3. Final assessment (T1): the participant undergoes the same clinical, biological, and instrumental investigations, as at T0, before discharge (approximately 8 weeks after admission).

**FIGURE 1:**
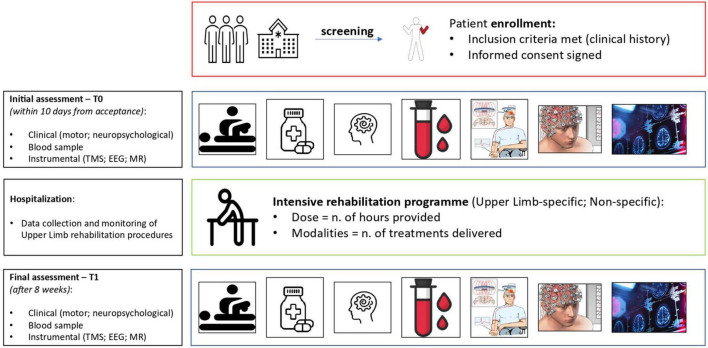
Schematic representation summarizing the different stages and the acquired measures of each participant involved in the NeuroPro study.

A full description of the clinical (motor and cognitive) assessments, blood sampling procedures, rehabilitation treatments (CT and OT) and devices employed, and instrumental investigations carried out in this study will be described below. See Figure 1 for a schematic representation of time and procedures employed for this project.

#### 3.2.4. Clinical motor profiling of recruited patients

Each participant recruited for the present study undergoes a detailed clinical assessment including: (1) collection of patient medical history and records; and (2) use of validated outcome measures for quantifying stroke severity, functional, and sensorimotor impairments.

The following outcome measures are used for quantifying the severity of stroke sequelae:

– National Institutes of Health Stroke Scale (NIHSS) (Page et al., 2012) is a 42-points scale for quantification of stroke severity.
– Oxfordshire Community Stroke Project (OCSP) (Bamford et al., 1991) is a clinical classification system for ischemic stroke, allowing to predict the site involved by the brain infarct.
– Functional Independence Measure (FIM) (Granger et al., 1986) is a 126-points scale for measuring the level of independence in activities of daily living (ADLs).

Following is the list of validated outcome measures for profiling UL sensorimotor impairments:

– Fugl-Meyer Assessment for Upper Extremity (FMA-UE) (Fugl-Meyer et al., 1975) is a 66-points scale for profiling UL impairment by quantifying performance of complex and segmental voluntary movements, grasping, and coordination. We will also the sensation and pain/range of motion domains of the FMA, with a total score obtainable of 0–24 and 0–48, respectively.
– Action Research Arm Test (ARAT) (Carroll, 1965) is a 57-points ordinal scale quantifying hand and arm activities.
– Medical Research Council (MRC) muscle strength scale (Compston, 2010) is a 5-points ordinal scale for assessment of voluntary force, applied to shoulder abduction (SA) and fingers extension (FE).
– Reaching Performance Scale (RPS) (Levin et al., 2004) is a 36-points scale for assessment of voluntary UL reaching task.
– Box and Blocks Test (BBT) (Mathiowetz et al., 1985a) is a 1-min test for assessment of gross manual dexterity.
– Nine Hole Pegboard Test (NHPT) (Mathiowetz et al., 1985b) is a 50-s test for assessment of fine finger dexterity.
– Trunk Control Test (TCT) (Collin and Wade, 1990) is a 100-points outcome measure for assessment of trunk control.
– Modified Ashworth Scale (MAS) (Bohannon and Smith, 1987) is an ordinal scale for assessment of muscle spasticity.

#### 3.2.5. Pharmacological treatment

Information on pharmacological therapy is also collected, both at admission and discharge. This is particularly relevant as many drugs are administered to patients after a stroke for treating associated symptoms (e.g., seizures, mood and behavioral disorders, pain, and sleep disorders). Those drugs are known to act on the central nervous system, thus potentially influencing the recovery process (Viale et al., 2018). The role played by specific types of drugs, such as antidepressant, benzodiazepine, antiepileptic, and neuroleptic, on recovery has been a major field of investigation in stroke survivors (Kumar and Kitago, 2019). In our study, drugs are categorized as antidepressants, antiepileptics, antipsychotics, benzodiazepine, and dopamine agonists according to their mechanism of action, with the aim to investigate any potential role in the recovery process after rehabilitation.

#### 3.2.6. Frailty index

Frailty is an emerging concept in the aging field that has proved to be of great clinical relevance. There are several ways of operationalizing frailty, but the most common (e.g., Fried’s method) is based on a composition of simple indices, mostly retrievable from clinical practice, such as unintended weight loss, reduced physical activity, motor slowness, in order to develop an actual score. Although this concept is important in the elderly (Rockwood and Howlett, 2018) it has been scarcely considered within rehabilitation settings where the patient is, most often, elderly and characterized by this very risk factor, which could potentially impact the recovery process.

#### 3.2.7. Quantification of rehabilitation intervention

Dose of therapy will be quantified both as the number of technological devices used for patient treatment (OT included) and dose (i.e., total number of hours) of intervention provided during the period of hospitalization. For the following analyses, dose of therapy is coded in terms of (i) total hours of rehabilitation specific for UL (i.e., UL technologies and plus OT), (ii) total hours of rehabilitation non-specific for UL (i.e., technologies for LL), and (iii) total amount of rehabilitation (i.e., hours of CT, and plus specific and non-specific UL interventions).

#### 3.2.8. Neuropsychological assessment

Every patient enrolled in the present study completes a detailed neuropsychological assessment before (T0) and after (T1) treatment evaluating the following cognitive and psychological domains [in brackets the tests used]:

– Global cognitive functioning [Oxford Cognitive Screen (OCS) (Mancuso et al., 2016; Bisogno et al., 2021) and Mini Mental State Examination (MMSE) (Folstein et al., 1975; Magni et al., 1996)];
– Abstract reasoning [Raven PM 47 (Carlesimo et al., 1996)];
– Attention and Executive functioning – visual search [Attentive Matrices (Della Sala et al., 1992)], visuospatial skills [Rey-Osterrieth Complex figure – copy (Caffarra et al., 2002)];
– Depression, anxiety levels [Depression Anxiety Stress Scale – DASS-21 (Lovibond and Lovibond, 1995)].

In addition to the above “core” assessment, specific protocols are used for right and left stroke patients.

Right stroke patients undergo to the following assessment:

– Attention and Executive function [B.I.T. Conventional (Wilson et al., 1987), Phonological fluency (Novelli et al., 1986), Modified Card Sorting Test (Kongs et al., 2000; Caffarra et al., 2004), Backward Digit Span (Monaco et al., 2013), Bell test (Mancuso et al., 2019)];
– Language [Designation on description and Semantic fluency (Novelli et al., 1986)];
– Memory – verbal short and long-term, verbal learning and spatial long term [Forward Digit Span (Monaco et al., 2013), Rey Auditory Verbal Learning Test - delayed recall (Carlesimo et al., 1996), Rey Auditory Verbal Learning Test – immediate recall (Carlesimo et al., 1996), Rey-Osterrieth Complex figure – delayed recall (Caffarra et al., 2002)].

Left stroke patients undergo to the following assessment:

– Attention and Executive functioning [Trail Making Test A and B (Giovagnoli et al., 1996; Tombaugh, 2004), Modified Card Sorting Test (Kongs et al., 2000; Caffarra et al., 2004), Modified-Five Point Test (Cattelani et al., 2011)];
– Memory – spatial short and long-term, spatial learning [Spatial Span forward (Spinnler and Tognoni, 1987), Rey-Osterrieth Complex figure – delayed recall (Caffarra et al., 2002), Spatial Supraspan (Spinnler and Tognoni, 1987)].

#### 3.2.9. Blood specimen protocol

Fasting blood samples are collected in EDTA tubes at T0 and T1. Plasma is removed and aliquoted for storage at −80°C until ready for analysis. Blood levels of a miRNA with potential predictive value (miR-941) (Edwardson et al., 2018) are quantified on samples acquired at T0. Similar analyses will be performed on samples acquired at T1 when appropriate, to investigate potential relationship with the study variables. The extraction and amplification of miRNAs are carried out with commercial kits as indicated by the manufacturer. Briefly, up to 800 μl of plasma is used for total RNA extraction with TRIzol LS Reagent (Thermo Fisher Scientific Inc., Waltham, MA, USA). RNA pellet is air-dried, resuspended in RNase-free water and stored at −80°C. Individual miRNA levels will be detected by real-time polymerase chain reaction (qRT-PCR), performed using the CFX96 Real-Time PCR detection System (Bio-Rad Inc., Hercules, CA, USA), with specific TaqMan MicroRNA Assay (Thermo Fisher Scientific n. 4427975). The expression level of miR-941 is normalized to miR-U6 snRNA as internal control.

### 3.3. Rehabilitation treatments

#### 3.3.1. Conventional therapy

The CT will consist of whole-body exercises selected autonomously by the clinician and performed one-to-one in a gym or a private room. For the UL, patients are asked to perform functional task exercises in each plane including shoulder and elbow flexion-extension, shoulder abduction-adduction, internal-external rotation, circumduction, and forearm pronation-supination. Moreover, exercises are proposed for training coordination and proprioception to stimulate the patient to enhance their residual capacities, reduce compensations, and control voluntary muscle activation. If needed, splints or orthosis can be considered (e.g., shoulder subluxation and spasticity of hand flexors). Each session lasts 1 h/day, 5 dd/w for each week of the hospitalization period, at minimum.

#### 3.3.2. Technological devices

Among all the modalities, also technologies for both upper and lower limb are available. Technologies for UL are: Virtual Reality Rehabilitation System (VRRS^®^, Khymeia Group Ltd., Noventa Padovana, Italy) consisting of a computer providing kinematic tasks displayed in a virtual scenario, to be emulated by patient’s real arm movement while controlling a virtual object, via motion tracking system; AMADEO^®^ (Tyromotion GmbH, Graz, Austria) an end-effector robot for the hand allowing to control selective hand opening/closing by means of electromyographic activities of wrist flexors and extensors; DIEGO^®^ (Tyromotion GmbH, Graz, Austria) a wired exoskeleton providing arm-weight support during virtual tasks; and REMO^®^ (Morecognition Ltd., Torino, Italy) a biofeedback armband for training of complex hand movements. Technologies for LL are: VRRS^®^ for LL and balance tasks; Gait Trainer (GT1^®^, Reha-Stim Medtec Inc., NY, USA) an end-effector robot providing body-weight support for walking training; Smart Balance Master^®^ (SBM, NeuroCom International Inc., Clackamas, OR, USA) an interactive balance platform for training exercises with visual biofeedback; OAK^®^ (Khymeia Group Ltd., Noventa Padovana, Italy) an integrated virtual reality system for assessment and prevention of risk of fall; Omego^®^ (Tyromotion GmbH, Graz, Austria) a multimodal robot for LL mobilization, muscle strength training, step initiating and trunk control. Each therapy with technologies is delivered 1 h/day, 5 dd/w, for 3 weeks, with a one-to-one (patient-specialized physiotherapist) approach. Number of repetitions, type of exercises could be settled by the physiotherapist according to clinical judgment and patient’s needs, tailoring difficulties on patient’s ability. All the technology-based modalities reported are included in the hospital clinical pathways and have been developed and validated through the institutional translational research projects funded by the Italian Ministry of Health and the European Commission, as described in previous publications (Beghi et al., 2018; Rimini et al., 2020; Baldan et al., 2021; Luque-Moreno et al., 2021; Salvalaggio et al., 2022; Pregnolato et al., 2023).

#### 3.3.3. Occupational therapy

During hospitalization patients may receive OT, consisting of UL-specific rehabilitation sessions targeted to ADLs (e.g., cooking, dressing, and washing), vocational (e.g., using a computer and writing), or recreational activities (e.g., sewing) important to them. OT could be delivered one-to-one, or in group sessions.

### 3.4. Physiological/imaging protocols

**FIGURE 2:**
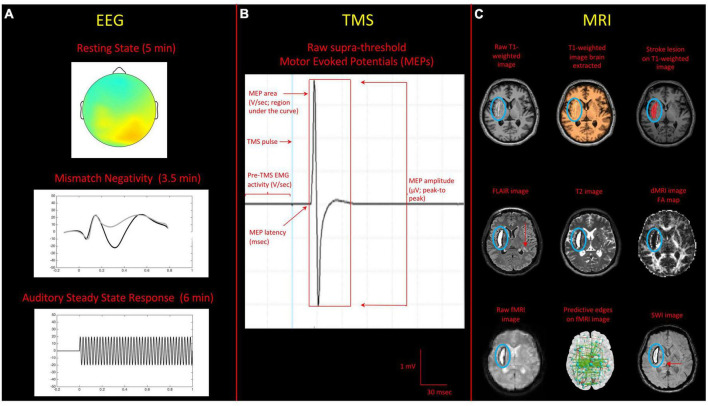
Summary of the acquired sequences for the physiological/imaging protocols implemented in the NeuroPro study. **(A)** EEG: the recordings represent standard protocols for clinical-experimental applications of EEG, widely used for prognostic studies. They include: resting-state (eyes open), Auditory Mismatch Negativity task, and an Auditory Steady State response investigating gamma (40 Hz) synchronization. **(B)** TMS: example of a prototypical motor evoked potential (MEP), indicating characteristics and measures of the main indexes of interest (i.e., MEPs amplitude, area, and latency). **(C)** MRI: images reported here on axial view include: raw T1-weighted image, T1-weighted image brain extracted, stroke lesion identified on T1-weighted image, FLAIR image, T2 image, fractional anisotropy (FA) map derived from diffusion MRI (dMRI) image, raw resting fMRI image, predictive functional connections from multivariate resting fMRI-behavior mapping (adapted from Calesella et al., 2021), susceptibility weighted image (SWI) in a representative participant. Red arrows on FLAIR and SWI image indicate the presence of deep white matter lesions and a black hole respectively. In all images the lesion area has been circled in blue.

For this project both physiological (i.e., EEG and TMS) and imaging (i.e., MRI) techniques are used. The synergistic use of EEG, MRI, and TMS will help to investigate convergent evidence at brain level, on the effect of rehabilitation after stroke. Moreover, the complementary use of neurophysiological non-invasive techniques may help to develop more sensitive markers for assessment of motor recovery. Traditional and novel Physiological/Imaging phenotypes will be derived from raw data and converted into interpretable features to be used in statistical models. Please see Figure 2 for physiological/imaging measures acquired.

#### 3.4.1. EEG protocol and data analysis

A 256-channel Geodesic EEG system (Magstim EGI Inc., Eugene, OR, USA) is used to record brain activity for each participant. During each session, each participant undergoes the following procedures: (1) 5 min resting state with eyes open, (2) 3 min Mismatch Negativity (MMN) task, with a passive auditory oddball, and (3) 6 min of Auditory Steady-State Response (ASSR). During the resting state, the participant is asked to sit and watch a fixation cross. When performing the MMN task, the patient listens to 240 tones characterized by a carrier frequency of 500 Hz (standard sounds), interspersed randomly with 60 tones with a 550 Hz carrier frequency (deviant sounds). The inter-stimulus interval (ISI) is set at 0.5 s and sounds are presented in a pseudorandom order such that the deviant sounds are always preceded by a standard sound. During this task, on the screen is projected the same fixation cross used for the resting state acquisition. Finally, during the ASSR recording, 180 trains of gamma auditory stimulation are delivered. Each stimulus lasts 1 s and with an ISI of 1 s. Gamma auditory stimulation is generated offline using the MATLAB software (2016b) and performed with 40 Hz amplitude-modulated (AM) tones, 1,000 Hz carrier frequency, 100% amplitude modulation and 6 ms fading in/out. Both MMN and ASSR tasks are delivered binaurally through earphones and presented with the freely available software PsychoPy Version 3.0.6 (Open Science Tools Ltd., Nottingham, UK^1^) (Peirce, 2007). At the end of the EEG recording session, neuronavigation of the patient’s head is performed with an iPad and the SPOT3D software (Taberna et al., 2019), in order to spatially localize EEG electrodes and match their position with the individual structural MRI.

Measures derived for each subject will include: (A) aperiodic parameters, such as broadband offset and exponent, extracted from the Power spectrum density data of the resting state recording, (B) event related potential’s (ERP) amplitude and latency (from the MMN task), and (C) frequency power over time obtained using a Morlet Wavelet transformation for each patient’s recording. These measures have been shown to be particularly sensitive when investigating stroke recovery (Lanzone et al., 2022) and cognitive dysfunction in neurological patients (Naatanen et al., 2011). Moreover, we will further explore the association between EEG-derived measures [i.e., Theta power (4–7 Hz) and Gamma power (39–41 Hz) over time] and severity of the pathology at T0 and to the degree of the recovery at T1 as recent evidence seems to suggest a potential association between these measures (Pellegrino et al., 2019).

#### 3.4.2. TMS protocol and data analysis

Transcranial magnetic stimulation (MagPro X100. MagVenture Inc., Alpharetta, GA, USA) is used in this project using a figure-of-eight coil (MC-B70. MagVenture Inc., Alpharetta, GA, USA). The most updated guidelines are followed in order to evaluate patients’ eligibility to TMS procedures (Rossi et al., 2021). Exclusion criteria include the presence of positive medical history for seizure or epilepsy, as well as the presence of heart disease or body-inserted devices. Study-participants wear a tissue cap where a grid of 1 cm-spaced points is drawn. Two self-adhesive disposable electrodes (Ag/AgCl) are placed on a tendon belly montage over *extensor digitorum communis* (EDC) muscle of the forearm, bilaterally (in addition to ground electrode). Electromyography (EMG) is recorded using a band pass filtering of 20–2,000 Hz (sampling rate: 5,000 Hz). TMS coil is always maintained on the scalp by the experimenter, at 45° with respect to the inter-hemispheric fissure, and with the handle pointing backwards. Firstly, the position on the scalp (*hot-spot*) that allows to obtain the highest and most reproducible MEPs from the contralateral EDC muscle is identified in the primary motor cortex, both in the left and the right hemisphere (participants at rest, open eyes). Thus, resting motor threshold (RMT) is bilaterally individuated as the stimulation intensity resulting in a MEP of at least 50 μV, in half of 8–10 consecutive trials, when stimulating the *hot-spot*. Resting state is always verified by EMG on-line visual inspection. Subsequently, 8–10 MEPs are recorded by stimulating the contralateral EDC motor representation at 120% of RMT (at rest, open eyes), in each hemisphere. In case RMT identification is not possible (e.g., absence of MEPs in the stimulated cortico-spinal pathway), the participant is asked to try to keep increasing levels of EDC muscular contraction, in order to verify MEPs presence or absence (thus allowing –or not– to record successive supra-threshold MEPs). For this reason, also 60 ms of pre-TMS EMG recordings are always obtained during acquisitions of supra-threshold MEPs, in order to verify muscular relaxation, or to refer the MEPs indexes to the pre-TMS EMG baseline activity. This setting is applied for every participant enrolled in the TMS procedures before the starting of the treatment (*baseline*) and at its end after the completion of the treatment.

Transcranial magnetic stimulation measures evaluated for this study include Motor Thresholds (i.e., RMT, see above) and supra-threshold MEPs. More specifically, RMT is defined as the percentage of the maximum possible stimulation intensity. Supra-threshold stimulation (obtained at 120% of RMT) is useful to obtain peak-to-peak MEP amplitudes (in μV), MEP areas (in V/s), and MEP latencies (in ms). Pre-TMS EMG is expressed in V/s.

#### 3.4.3. MRI protocol and data analysis

Scanning is carried out at the IRCCS San Camillo Hospital, Venice using a 3T Ingenia Scanner (Philips Inc., Amsterdam, Netherlands) with a 32-channel receive head coil. The neuroimaging protocol comprises both structural and functional sequences and lasts approximately 40 min. MRI sequences include (a) high-resolution T1-weighted, (b) diffusion MRI (dMRI), (c) resting-state functional MRI (rs-fMRI), (d) fluid attenuated inversion recovery (FLAIR), (e) T2, and (f) susceptibility weighted imaging (SWI). A description of the MRI parameters adopted here is provided in Table 1. Data analysis will be performed using FSL (FMRIB Software Library), statistical parametric mapping (SPM), Free-Surfer, and other available packages and in-house developed tools. Participants with contraindications to MRI scanning (including but not limited to a history of claustrophobia, certain metallic implants, and metallic injury to the eye) are excluded from the study.

**TABLE 1:** Brain MRI sequences and scanning parameters used in the NeuroPro study.

Sequence type	T1w MPRAGE	T2w TSE	FLAIR	SWI	GE EPI (fMRI)	DTI_MS_AP/B0_PA (for DTI distortion correction)	SE EPI (for fMRI distortion correction)
Orientation	Sagittal	Sagittal	Sagittal	Transverse	Transverse	Transverse/“”	Transverse/“”
TR (ms)	6.8	3,000	8,000	31	1,000	3,700/“”	1,900/“”
TE (ms)	3	280	360	7.2/13.4/19.6/25.8	23	104/“”	60/“”
Flip angle (degree)	8	90	90	17	50	90/“”	90/“”
Resolution (mm^3^) (FH, AP, RL)	1 × 1 × 1	1 × 1 × 1	1.12 × 1.12 × 1.2	2 × 0.6 × 0.6	3 × 3 × 3	2 × 2 × 2/“”	3 × 3 × 3/“”
FOV (mm^3^) (FH, AP, RL)	240 × 240 × 181	240 × 240 × 190	247 × 247 × 183	130 × 230 × 199	168 × 240 × 240	156 × 224 × 224/“”	168 × 240 × 240/“”
Slices	–	–	–	–	56	78/“”	56/“”
Water fat shift (pixels)	1.6	0.73	0.73	1.7	10.3	15.6/“”	10.4/“”
EPI factor	–	–	–	–	51	59/“”	53/“”
SENSE acceleration	No	1.8 AP, 2 RL	No	3 RL, 1.5 FH	1.55	1.9/“”	1.5/“”
Compressed SENSING acceleration	3.5	No	8	No	–	–/“”	–/“”
Multiband acceleration	–	–	–	–	4	3/“”	No
*b*-Values	–	–	–	–	–	0, 300, 1,000, 2,000/0	0/0
Volumes/NSA/b-directions	1	1	2	1	600 (6 dummies)	12, 8, 32, 64/10	1 (6 dummies)/1 (6 dummies)
Acquisition time	3 min 14 s	4 min 30 s	5 min 36 s	2 min 41 s	10 min 9 s	7 min 18 s/45 s	46 s/“”

*(A) T1-weighted*: this sequence is primarily used to study gray matter (GM) structural macroscopic tissue in both cortical and subcortical brain regions. GM changes have been widely reported in stroke patients (Diao et al., 2017) and associated with motor recovery (Abela et al., 2015). Brain tissues can be segmented into total GM, WM and cerebrospinal fluid (CSF), and cortical and subcortical regions. Brain tissues and (sub)-cortical regions will be visually inspected to ensure an accurate segmentation. T1-weighted images will also be used to carry out the lesion segmentation procedure (i.e., the reconstruction of individual patient’s lesion subsequent the stroke event). The identification of 3D lesion maps for all the recruited patients is a necessary step for subsequent processing and analysis not only of the acquired MRI data, but also for the neurophysiological data.

*(B) Diffusion MRI (dMRI)*: diffusion MRI exploits the principles of traditional MRI to measure the random motion of water molecules and subsequently to (I) infer information about WM microstructural properties and (II) delineate the gross axonal organization of the brain (Bammer, 2003). As dMRI is particularly sensitive to susceptibility-induced distortions, here we have adopted a correction strategy based on the complementary information from pairs of diffusion images acquired with reversed phase-encoding (PE) directions to correct for distortions. Moreover, a multi-shell acquisition was specifically implemented for this project, which will allow to account for crossing fibers issues and will provide a higher resolution for the intravoxel structure. These aspects are crucial when attempting to accurately reconstruct WM bundles in the presence of lesions and assess how micro-structural connectivity can be affected by stroke and modulated by rehabilitation (Konieczny et al., 2021). FA, mean diffusivity (MD), axial diffusivity (AD), and radial diffusivity (RD) maps, known to be sensitive to brain insults, such as stroke, can be generated. Individual matrices reflecting structural connectivity will be derived and used as predictors of motor recovery (Rocha et al., 2022).

*(C) Resting state functional MRI (rs-fMRI)*: rs-fMRI is used to investigate resting state networks (RSNs), which encompass brain regions with a common time-course of spontaneous fluctuations and reflecting properties of functional brain organization (Smith et al., 2009). The main advantage of this approach, compared to task-based fMRI, is that it is data-driven, therefore does not rely on any *a priori* hypothesis, any explicit temporal model, or the choice of a specific task, which may be difficult to match between subject groups, particularly in clinical studies, where the correct understanding of instructions may be affected by the patient cognitive status. Changes in RSNs have been widely reported to define functional changes in stroke patients (Ovadia-Caro et al., 2014) and recent studies have highlighted their potential utility to predict recovery after stroke (Park et al., 2011). All study-participants are instructed to lie in dimmed light with their eyes open, blink normally, but not to fall asleep. In order to reduce images artifacts, the same correction method described for the DTI data will also be applied to rs-fMRI images. Data analysis will be performed to derive individual connectomics matrices (Calesella et al., 2021).

*(D–E) Fluid attenuated inversion recovery (FLAIR) and T2*: both these sequences are commonly used in clinical practice to characterize stroke-induced lesions, periventricular lesions adjacent to the sulci, WM hyperintensities, and WM lesions (Haller et al., 2013).

*(F) Susceptibility-weighted imaging*: SWI images are particularly sensitive to compounds which distort the local magnetic field and as such they are useful in detecting blood products, iron and calcium, which are a common result of brain insults, such as stroke (Hermier and Nighoghossian, 2004).

### 3.5. Statistical modeling

Statistical methods will be based on the intention-to-treat principle (McCoy, 2017). Given the heterogeneity of the measures collected, data will be normalized before being analyzed and in case of high percentages of missing data, they will be subjected to multivariate imputation (Sterne et al., 2009). The statistical analysis will be as follow:

– Descriptive statistics (median, interquartile range, mean, standard deviation, and absolute and percentage frequencies) will be applied to describe the sample characteristics and data.
– Data distribution, assessed with the Shapiro–Wilk test, and appropriate correlation tests (i.e., Spearman’s rho, Pearson’s *r*, Cramér’s V, or point-biserial correlation coefficient) will be performed to study the presence of associations between the variables (collinearity).
– Data grouping. Patients will be divided in two categories (i.e., *Responders* and *Non-Responders*) according to responsiveness to therapy, defined as an improvement of 5 points relative to the minimally clinically important difference (MCID) of the primary outcome measure (i.e., FMA-UE) (Page et al., 2012). To assess whether there is a statistically significant difference in the dose of therapy between the Responder and Non-Responder patient groups, Student’s *t*-test for unpaired data or Mann–Whitney test for each clinical variable will be performed, depending on distribution properties, as already proposed in previous studies (Salvalaggio et al., 2023). Then, with the aim to identifying the best set of variables (regressors) that can predict UL motor recovery in patients, appropriate regression models will be estimated. This step will be preceded by the evaluation of the requirements of the chosen model and by considering possible adjustments for mediating, moderating, and confounding factors. Prior to model fitting, preliminary diagnostic check will be made [i.e., variance inflation factor (VIF) for the study of collinearity independence among predictors]. Selection of the best set of regressors will be made taking into account appropriate indices. A diagnostic analysis (goodness-of-fit) of the model will be performed on the model to verify its assumptions and the fit to the available data. The statistical significance level will be set at *p* < 0.05, and all statistical analyses will be performed using the latest version of R software (R Core Team, 2022).

## 4. Discussion and summary

The identification of sensitive measures, which allows a deep investigation of neural processes underpinning recovery after focal injury, is a current need, particularly in clinical settings (Bernhardt et al., 2016; Hayward et al., 2017).

The study described here will generate a rich multi-modal and integrated dataset in stroke patients, characterized by a wide range of complex measures derived from traditional and advanced assessment modalities. This is an essential step both to confirm acknowledged predictive models and to develop new ones for a careful evaluation of UL functional recovery following rehabilitation. The identification of a set of markers, able to finely predict the UL rehabilitation induced sensorimotor recovery after stroke, could be particularly useful for patients’ stratification (e.g., *Responders* and *Non-Responders*, according to patients who overcome the MCID in clinical scales) (Dimyan et al., 2022). This will contribute to steer the actual rehabilitation practice toward a more personalized treatment (or combination of treatments), and therefore reach the best possible outcome at a functional and sensorimotor level.

This study is not without limitations and challenges. Indeed, the use of numerous outcome measures and evaluation methods employed here require a careful statistical planning and modeling, also considering the potential of missing data. Moreover, the lack of a control cohort may limit the generalizability of “candidate” prognostic factors in terms of causal relationship between predictors and final outcomes (Kent et al., 2020). For this reason, we envisage, as a possible extension of the present study, the external validation of the identified model(s) using a randomized controlled trial (RCT) or a cost-effectiveness study, which will eventually provide the possibility to guide the process of clinical decision-making regarding the time of intervention, promoting the greatest chance of recovery of the compromised functions.

## Ethics statement

Ethical approval was granted by the “Comitato etico per la Sperimentazione Clinica (CESC) della Provincia di Venezia e IRCCS San Camillo” (Prot. 1375/IRCCS San Camillo). The patients/participants provided their written informed consent to participate in this study.

## Author contributions

SS and AT devised and planned the study and wrote part of the first draft of this manuscript. DM and GA contributed to the funding. SS, MA, EM, and AV screened and enrolled patients and collected the motor data. GF designed the imaging protocol. NF wrote part and critically revised the final draft of this manuscript. AC and IP contributed to the medical clinical protocol and wrote part of this draft. FB, LD, RP, and ER wrote part of this draft and planned the neuropsychological protocol. RB planned the blood sample acquisition. PB and SZ wrote part of this draft and planned the TMS protocol. GA and SZ wrote part of this draft and planned the EEG protocol. DD’I and MZ wrote part of this draft and prepared the pipeline for the lesion segmentation. LM wrote part of this draft and contributed to the statistical analysis planning. All authors contributed to the data acquisition and analysis, read and approved the final manuscript.

## Footnotes

1
http://www.psychopy.org/
